# Nanomedicine in the Diagnosis and Treatment of Pancreatic Cancer

**DOI:** 10.3390/pharmaceutics17040449

**Published:** 2025-03-31

**Authors:** Kexin Guo, Sicheng Li, Xinyu Wu, Huihua Xiong

**Affiliations:** Department of Oncology, Tongji Hospital, Tongji Medical College, Huazhong University of Science and Technology, Wuhan 430030, China; m202476582@hust.edu.cn (K.G.); lisicheng19@163.com (S.L.)

**Keywords:** pancreatic ductal adenocarcinoma, nanomedicines, nanoprobe, signaling pathways

## Abstract

Pancreatic ductal adenocarcinoma (PDAC) is an aggressive malignancy with increasing incidence and mortality rates, highlighting the urgent need for early diagnosis and treatment. However, early diagnosis of PDAC is extremely challenging due to the atypical early symptoms or the absence of noticeable symptoms. As a result, many patients are diagnosed with local metastasis, and even patients who are eligible for surgical resection have a high postoperative recurrence rate. Consequently, chemotherapy remains the primary treatment for PDAC. However, the unique biological characteristics of PDAC not only promote tumor progression and metastasis but also often lead to chemoresistance, a significant barrier to successful treatment. Recently, nanomaterials have garnered significant attention as promising materials for diagnosing and treating PDAC, showing great potential in cancer therapy, imaging, and drug delivery. Novel targeted nanomedicines, which encapsulate chemotherapy drugs and gene therapy products, offer significant advantages in overcoming resistance. These nanomedicines not only provide innovative solutions to the limitations of conventional chemotherapy but also improve the selectivity for cancer cells to enhance therapeutic outcomes. Current research is focused on the development of advanced nanomedicines, such as liposomes, nanotubes, and polymer-lipid hybrid systems, aimed at making chemotherapy more effective and longer lasting. This review provides a detailed overview of various nanomedicines utilized in the diagnosis and treatment of PDAC and outlines future directions for their development and key breakthroughs.

## 1. Introduction

### 1.1. Introduction of Nanomedicines

Recently, the rising incidence and mortality rates of cancer have posed significant challenges to global public health, making its diagnosis and treatment critical issues. Nanomedicine (NPs) has been applied in cancer diagnosis and treatment due to its advantages, including strong targeting capability, high drug stability, and the ability to load a variety of materials. Compared to traditional drugs, NPs are smaller in size and enable specific cancer biomarker detection in a simple and non-invasive manner, offering multiple advantages in early tumor diagnosis due to their enhanced sensitivity. Additionally, due to the enhanced permeability and retention (EPR) effect, NPs preferentially accumulate in tumor tissues, improving their diagnostic and therapeutic efficacy and specificity [1,2]. Moreover, by loading biomolecules, such as DNA, RNA, peptides, or antibody fragments, onto the surface of NPs, they can be selectively internalized in certain tumor cells through specific or overexpressed receptors while minimizing toxicity to normal cells [3]. Therefore, more advanced drugs targeting different types of NPs (such as liposomes, micelles, polymeric nanoparticles, and inorganic nanoparticles) are currently under development and being applied in the diagnosis and treatment of PDAC. In the following sections, we introduce different NPs that have been applied in the diagnosis and treatment of PDAC.

### 1.2. Challenges in Diagnosis and Treatment of PDAC

In recent years, cancer, the second leading cause of death in the United States, has attracted global attention for its diagnosis and treatment [4]. Pancreatic cancer is a highly aggressive malignancy and the fourth leading cause of cancer-related deaths. More than 90% of pancreatic cancers are PDAC, which has a five-year survival rate of less than 10% [5]. Due to its insidious onset, subtle symptoms, and lack of specific biomarkers, early diagnosis is often challenging. The clinically used marker carbohydrate antigen 19-9 (CA19-9) has a sensitivity of 76.1%; however, it lacks tumor specificity, as elevated levels can also be observed in various malignancies and other conditions (such as liver damage, bile duct obstruction, and pancreatitis) [6,7,8]. Additionally, imaging techniques, like computed tomography (CT), magnetic resonance imaging (MRI), and positron emission tomography (PET), are often utilized in the diagnosis of PDAC. CT and MRI are commonly used imaging modalities, and their sensitivity is correlated with tumor size. For tumors smaller than 20 mm in diameter, the first-line imaging modality, CT, has limited sensitivity and may not offer significant clinical assistance in the early diagnosis of PDAC [9]. Therefore, identifying more specific and sensitive tumor markers or imaging techniques has become a key focus of current research.

Since the difficulty of early diagnosis, many patients are diagnosed with local metastasis, and less than 20% of patients are eligible for surgical resection, with a postoperative recurrence rate as high as 71% [7]. Therefore, chemotherapy remains the primary treatment modality for PDAC. However, the inherent chemoresistance of PDAC and physical barriers to drug delivery significantly contribute to chemotherapy resistance. Several hereditary or acquired gene mutations, like *KRAS*, *TP53*, and transforming growth factor beta (*TGF-β*), play an important role in this unfavorable prognosis. Additionally, a defining feature of PDAC is its dense tumor stroma, which can constitute over 90% of the total tumor volume and plays a major role in tumor progression and chemoresistance. The stroma microenvironment comprises cancer-associated fibroblasts (CAFs), pancreatic stellate cells (PSCs), endothelial cells, and immune and endocrine cells [10]. The fibrotic barrier increases interstitial pressure and reduces micro-vessel density, thereby impeding drug penetration. Tortuous and low-permeable vessels lead to the uneven distribution of medicines in tumor tissues, and the tight junctions of vascular endothelial cells also lead to a decrease in drug permeability in tumor tissues [11]. Moreover, stromal components such as CAFs promote tumor progression by secreting pro-tumor cytokines. Additionally, this persistent tumor escapes immune detection through the secretion of immunosuppressive factors, the expression of immune checkpoints, and the creation of low T lymphocytes [12]. In summary, the tumor microenvironment (TME) in PDAC—characterized by high pressure, hypoxia, acidic pH, immune evasion, and a fibrous stroma—promotes rapid tumor proliferation, metastasis, and invasion, leading to poor prognoses associated with conventional therapies, leading to obstacles in the delivery of drugs and necessitating the need for further research to address these challenges.

## 2. The Diagnosis of PDAC Based on Nanomedicine

**Figure 1:**
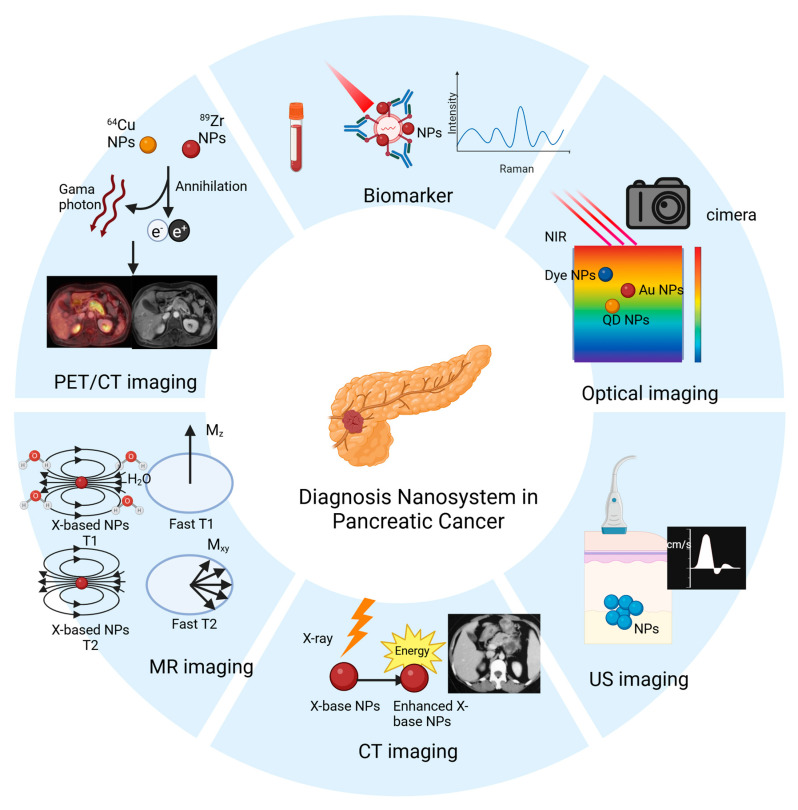
Applications of nanomaterials in the diagnosis of PDAC, including nanomaterial-based in vitro detection techniques, and nanomaterial-enabled in vivo imaging techniques (e.g., optical imaging, ultrasound (US) imaging, CT imaging, MR imaging, and PET/CT imaging).

As PDAC is a highly malignant tumor, there are no specific tumor biomarkers or imaging techniques for its diagnosis. In recent years, with the advancement of nanotechnology, targeted biomarkers, and nanomedicines, including those used as imaging agents, have shown considerable success in the diagnosis of PDAC (Figure 1). In this section, we discuss the different nanomedicines applied in laboratory detection and imaging techniques.

### 2.1. Biomarkers Detection Based on Nanomedicine

As an important method for the early diagnosis of cancer, laboratory detection remians at the forefront of development. In this section, we introduce nanomedicines based on different biomarkers for sensitive and specific diagnosis of pancreatic cancer (Table 1).

#### 2.1.1. Detection of Biomarkers in Body Fluids

Body fluids are readily accessible biological materials that are essential for disease diagnosis. Various nanomedicines have been developed to enhance the sensitivity and specificity of PDAC diagnosis. CA19-9, a commonly used blood biomarker for PDAC diagnosis, exhibits limited sensitivity. To improve detection accuracy, Huang et al. used gold nanoparticles (AuNPs)@PThi nanomedicine. When immobilized on a a glassy carbon electrode, it functions as a sensitive redox probe for the electrochemical detection of CA19-9. This approach enabled ultrasensitive detection across a concentration range of 6.5–520 U/mL, with a calculated detection limit of 0.26 U/mL [13].

In addition to CA19-9, other blood protein markers, such as carcinoembryonic antigen (CEA), are commonly used to diagnose PDAC. For instance, Liu et al. developed an enzyme-labeled AuNP probe. By encapsulating AuNPs with antibodies, single-stranded DNA (ssDNA), and horseradish peroxidase (HRP), the nanoparticles achieved sensitive and specific detection of CEA with a detection limit of 12 ng/L in human serum, which is approximately 130 times more sensitive than conventional ELISA [14]. Similarly, Krasnoslobodtsev et al. utilized AuNPs as a potential biomarker for detecting overexpressed mucin MUC4 in the blood of patients with PDAC through the surface-enhanced Raman scattering (SERS) effect, enabling the highly sensitive detection of a single AuNP [15].

In addition to tumor-specific proteins, alterations in nucleic acid expression levels can serve as predictive markers for tumor occurrence. Compared to non-malignant cells, microRNAs (miRs) such as miR-10b, miR-21, miR-155, and miR-196a were significantly upregulated, while miR-31 and miR-357 were significantly downregulated in PDAC cells, facilitating the development of diagnostic tools [24]. Leveraging this, Joshi et al. developed an AuNP with a localized surface plasmon resonance (LSPR) sensor to detect miR levels in the plasma of patients with PDAC. This method allows for the simultaneous measurement of multiple miRs while differentiating PDAC from chronic pancreatitis patients [16]. Furthermore, Xu and Liao group utilized an α-hemolysin (αHL) single nanopore to detect miR-21 and distinguish complex molecular signals of miR-21, miR-155, and miR-196a, providing new PDAC diagnosis insights [17].

Extracellular vesicles (EVs), a heterogeneous group of cell membrane structures associated with tumor progression and metastasis, are potential biomarkers [25]. Rodrigues et al. developed a semiconductor nanomaterial-based rapid fluorescence immunoassay to normalize EV biomarker levels to EV abundance. This approach enabled the detection of the expression of two pancreatic cancer biomarkers, live erythropoietin-producing hepatocellular A 2 (EphA2), and epithelial cell adhesion molecule (EpCAM) [18].

Lewis et al. developed an alternating current (AC) electrokinetic microarray chip capable of directly identifying and quantifying Evs in whole blood, serum, or plasma. The biomarkers glypican-1 and CD63 could distinguish 20 PDAC patient samples from 11 healthy controls with 99% sensitivity and 82% specificity [19]. Similarly, Li et al. designed a polydopamine (PDA)-modified slide that encapsulated antibodies targeting exosome surface proteins (anti-macrophage migration inhibitory factor (MIF),anti-Glypican1 (GPC1), anti-CD63, or anti-epidermal growth factor receptor (EGFR)). By capturing exosomes from samples and forming a “chip-exosome-PDA encapsulated antibody-reporter-Ag (shell)–Au (core) multilayer (PEARL) label” complex, this system enables ultra-sensitive detection of metastatic and non-metastatic PDAC through Raman spectroscopy, differentiation stage I-II from stage III tumors without histopathological examination [20].

Urine, an easily obtainable and storable body fluid, also offers the potential for PDAC detection. Liu et al. investigated a nanobiotinylated liposome based on isothermal amplification (LAMP) to detect urinary Regenerating Family Member 1 Alpha (REG1A), a protein biomarker for PDAC. Compared with polymerase chain reaction (PCR), LAMP is simpler, faster, and more specific, with a detection limit of 1 fg/mL [21].

#### 2.1.2. Detection of Biomarkers in Cells and Tissues

In addition to body fluids, abnormally expressed antibodies or proteins in tumor cells and tissues are commonly used for early tumor detection. Optical nanoprobes have emerged as promising diagnostic tools. For example, Gisela et al. developed a sensor for CA19-9 detection by immobilizing anti-CA19-9 antibodies on screen-printed interdigitated electrodes (SPIDEs). The device achieved a CA19-9 detection limit of 0.12 U/mL [22].

To detect another PDAC biomarker, claudin-4, Hwang et al. constructed nanoprobes based on modified apoferritin (mAFTN) and functionalized CdSe (ZnS) quantum dots (QDs). This nanomedicine showed fluorescence detection sensitivity 27 times higher than that of conventional organic fluorophores and six times higher than that of individual QDs [26].

Eck et al. synthesized AuNPs with heterobifunctional polyethylene glycol (PEG) and covalently linked them to a F19 monoclonal antibody targeting fibroblast activation protein (FAP), which is highly expressed in PDAC-reactive stromal fibroblasts. The resulting conjugates successfully labeled tumor stroma in approximately 5 µm thick excised PDAC tissue slices and were imaged using dark-field microscopy at around 560 nm resonance scattering [23].

### 2.2. Imaging Examination Based on Nanomedicine

In addition to the laboratory detection of tumor biomarkers, radiological techniques are crucial for the early diagnosis of PDAC. In contrast, conventional imaging methods detect tumors only when they exceed 1 cm in diameter, with results highly relying on factors such as the operator’s experience and the patient’s condition [23]. To overcome these limitations, the development of nanomedicine-based imaging approaches has become a promising research direction aimed at enhancing diagnostic sensitivity and specificity.

When integrated with imaging modalities such as near-infrared (NIR) fluorescence, MRI, CT, PET, US imaging, and photoacoustic imaging (PAI), nanoplatforms offer prolonged circulation times and improve tumor targeting through active or passive NPs modification (Table 2). These systems play a prominent role in drug delivery monitoring, treatment response assessment, and image-guided therapy [27].

#### 2.2.1. NIR Imaging

NIR fluorescence imaging, operating in the range of 700–900 nm, offers superior contrast and deeper tissue penetration compared to visible light, and has attracted much attention for the early diagnosis of PDAC [35]. The combination of nanomedicines with NIR dyes has emerged as a promising research avenue.

For instance, Li et al. developed silica nanoparticles (bMSN@Cy7.5-FA NPs) by conjugating the NIR dye Cy 7.5 with folic acid (FA), which can be used for in vivo tumor visualization with peak fluorescence intensity within 12 h, providing an effective platform for early PDAC diagnosis [28]. Similarly, the croconaine (CR) dye has shown potential for expanding NIR applications. Dong et al. constructed a photothermal molecular delivery system (CR@E8-EVs) by conjugating Cadherin 17 (CDH17) nanobodies with Evs. This system exhibits strong NIR absorption, excellent photothermal properties, good biocompatibility, and remarkable active tumor-targeting capability [29].

Han et al. combined GEM with the NIR dye IR780 to form human serum albumin (HSA)-GEM/IR780 complexes, which showed enhanced tumor accumulation and retention, with detectable fluorescence signals persisting up to 72 h post-injection. This extended retention is promising, since most cyanine NIR probes exhibit rapid degradation in vivo [36]. One plausible explanation for this phenomenon is defective lymphatic drainage in tumor tissues, which reduces the clearance of nanomedicines and prolongs their local retention [37].

Targeting the overexpressed cholecystokinin B (CCK-B) receptor in PDAC, AP1153 shows high specificity and affinity (15 pM), nearly three orders of magnitude greater than that of its peptide ligand, gastrin [30]. Building on this, Abraham et al. conjugated AP1153 with indocyanine green (ICG) to form AP1153-ICG-NJs, achieving peak fluorescence at 18 h post-injection for highly specific early PDAC detection [38]. Likewise, Clawson et al. synthesized amorphous calcium phosphate silica nanoparticles (CPSNP) that covalently coupled ICG with AP1153, achieving tumor fluorescence peaks between 15 and 18 h without stimulating PDAC cell proliferation, further facilitating early PDAC diagnosis [30].

#### 2.2.2. CT Imaging

Enhanced CT imaging is a conventional adjunctive tool for the early diagnosis of PDAC. However, the short circulation half-life of iodine-based contrast agents necessitates precise control of the injection speed and timing during the examination. In contrast, stable nanoparticles ranging from 10 to 500 nm exhibit prolonged blood circulation and can accumulate at tumor sites through active targeting, offering distinct advantages for early PDAC detection [39,40].

Inorganic nanomaterials, including gold (Au), bismuth (Bi), and platinum (Pt), are considered potential alternatives for contrast enhancement. Trono et al. designed AuNPs for PDAC imaging, showing preferential uptake by PDAC cells at an optimal diameter of 20 nm. In addition to metallic nanoparticles, Xu et al. employed bismuth subcarbonate nanotubes (BNTs) for tumor-targeted imaging. BNTs not only provide superior contrast compared to conventional contrast agents but also exhibit therapeutic potential by suppressing tumor volume through combined radiotherapy and chemotherapy [41].

#### 2.2.3. MR Imaging

MRI is commonly used for clinical diagnosis, especially for detecting small cystic lesions. However, its sensitivity in identifying PDAC remains limited, hindering early diagnosis. To address this issue, Holbrook et al. designed a Gd(III) contrast agent (Lip-Gd@AuNPs) that can accumulate in pancreatic tissue, providing significant signal enhancement. By allowing clear detection of the pancreas with a contrast ratio exceeding 35:1, this method contributes to the early diagnosis of PDAC [32].

Biocompatible superparamagnetic iron oxide nanoparticles (SPIONs) have also been explored as MRI contrast agents, offering reduced toxicological risk and avoiding nephrogenic systemic fibrosis (NSF) [42]. He et al. developed a chemokine receptor 4 (CXCR4)-targeted ultrasmall superparamagnetic iron oxide (CXCR4-USPIO) for PDAC imaging. Their findings showed that the T2 enhancement ratio and ΔR2 values could semi-quantitatively assess CXCR4 expression in four pancreatic cancer cell lines (AsPC-1, BxPC-3, CFPAC-1, and PANC-1), potentially serving as prognostic indicators [33].

In another study, Zhou et al. designed ultrasmall superparamagnetic iron oxide nanoparticles (USPIO-NPs) that could serve as MRI contrast agents or be cross-linked with NIR dyes to achieve dual MR/NIR imaging [43]. Wang et al. designed superparamagnetic iron oxide NPs (Dex-g-PCL/SPIO nanoparticles) targeting enolase 1 (ENO1), a protein upregulated in PDAC. Both in vitro and in vivo MRI studies have demonstrated enhanced detection efficiency for PDAC [34].

#### 2.2.4. PET Imaging

PET/CT, as an emerging diagnostic method, is increasingly used for tumor diagnosis and metastasis monitoring due to its high sensitivity and specificity. However, conventional contrast agents like 18F-fluorodeoxyglucose (18F) exhibit limited sensitivity for PDAC detection and are prone to false-positive results under inflammatory and hyperglycemic conditions, reducing their suitability for early diagnosis [44]. In contrast, some nanoparticle-based contrast agents not only minimize false-positive results but also offer enhanced specificity for PDAC, making them a promising tool for early diagnosis.

For example, Chakrabarti et al. designed a hybrid nanoprobe ([^64^Cu]KRAS-IGF1) based on the commonly mutated gene, Kirsten rat sarcoma viral oncogene homolog (*KRAS*), in PDAC. After four hours post-injection, the PDAC signal intensity was 8.6 ± 1.4 times more intense than that of the contralateral muscle [45]. Additionally, Reiner et al. constructed nanoparticles ([^89^Zr]-NRep) by conjugating Doxil with zirconium-89, which accumulate in tumor tissues regardless of size and enable precise quantitative measurement of Doxil distribution within the tumors [46].

## 3. The Treatment of PDAC Based on Nanomedicine

**Figure 2:**
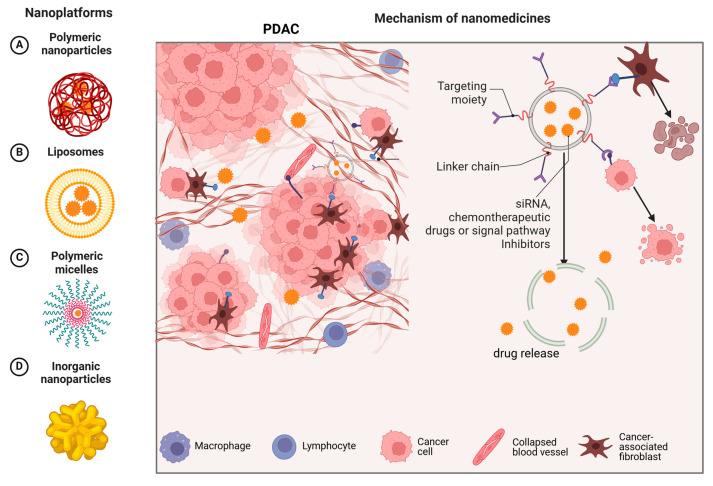
The left side A, B, C, and D show different types of nanomedicine delivery systems. The right side explains how these nanoplatforms operate, emphasizing the targeting moiety, linker chain, and therapeutic components (such as siRNA, chemotherapeutic drugs, or signal pathway inhibitors) that work together to enhance the treatment efficacy.

The robust desmoplastic stroma and immunosuppressive TME of PDAC hinder effective drug delivery, leading to chemoresistance. Nanomedicines, due to their small size and EPR effect, can be combined with therapeutic molecules to achieve effective accumulation at the tumor site while reducing toxicity to surrounding cells. Many advanced drugs targeting different types of NPs (such as liposomes, micelles, polymeric nanoparticles, and inorganic nanoparticles) are currently under development and are being applied in the treatment of PDAC (Figure 2).

In this section, we introduce four types of nanomedicines for the treatment of PDAC (Table 3). Each category of nanomedicine is discussed in detail concerning chemotherapy, immunotherapy, targeted therapy, and other relevant approaches.

### 3.1. Polymeric Nanoparticle

Polymeric nanoparticles are particles with a size ranging from 1 to 1000 nm, which can encapsulate, embed, or conjugate active compounds and are capable of carrying multifunctional agents [67]. Known for their biocompatibility and biodegradability, they can conjugate, encapsulate, and carry various drugs or materials. Compared to conventional colloidal systems, they offer several advantages, including reduced toxicity, prolonged circulation times, enhanced cellular uptake via the EPR effect, improved bio-stability, uniform organ distribution, and controlled drug delivery [68].

#### 3.1.1. Targeted Therapy Drugs

siRNAs are a crucial component of targeted therapy and have been extensively utilized in nanoparticle-based approaches for PDAC. CAFs are the predominant component of the PDAC stroma and contribute to PDAC progression through pro-tumor signaling and fibrosis production. Thus, the inhibition of CAFs is a promising therapeutic strategy [69]. SLC7A11 is a cystine transporter primarily expressed in CAFs that promotes their proliferation. Inhibiting its expression can reduce both CAF proliferation and PDAC proliferation and metastasis. Sharbeen et al. investigated Star3 nanoparticles complexed with SLC7A11 siRNA (Star3+SLC7A11-siRNA), which effectively downregulated SLC7A11 in PDAC mice, thereby reducing tumor growth, metastasis, and intratumoral fibrosis [47].

βIII-tubulin, which is linked to *KRAS* mutations, is overexpressed in PDAC tissues but is absent in normal acinar and ductal cells, facilitating cancer cell growth and metastasis [70]. Lo et al. designed a tandem peptide delivery system incorporating *KRAS* siRNA with an internalizing RGD peptide (iRGD)-targeting peptide. This construct successfully silenced the target gene in PDAC cell lines and immune-active genetically engineered mouse models, achieving significant therapeutic effects in 3D models [48]. Teo et al. developed star-poly (dimethylaminoethyl methacrylate) (POEGMA), which readily self-assembles with siRNA to form nanoparticles. Systemic administration of these nanoparticles in an orthotopic pancreatic tumor model resulted in high siRNA accumulation and silenced 80% of βIII-microtubule expression at both the gene and protein levels, thereby inhibiting tumor growth and metastasis [71].

MiR-21 targets and inhibits several tumor suppressor genes, including Programmed cell death protein 4(PDCD4), Phosphatase and tensin homolog deleted on chromosome ten (PTEN), and Tropomyosin 1 (TPM1) [72]. Li et al. provided a miR-21 antisense oligonucleotide (ASO-miR-21) combined with GEM and polyethylene glycol-polyethylenimine, which inhibited PDAC cell proliferation and metastasis [73]. Mutations in *TGF-β* are prevalent in patients with advanced PDAC and are correlated with poor survival. Ellermeier et al. The use of polyethylenimine (PEI)-coated delivery of TGF-β1 siRNA in an orthotopic PDAC model significantly reduced tumor TGF-β expression and prolonged mouse survival [49].

PRDM14 is overexpressed in various cancers but is undetectable in normal tissues and promotes PDAC metastasis. Taniguchi et al. developed a branched polyethylene glycol-conjugated poly-(l-ornithine) (PEG-PLO) nanoparticle for delivering PRDM14-targeted siRNA, achieving tumor-specific accumulation and effectively reducing tumor size and metastasis in vivo [73]. Similar outcomes were observed with calcium phosphate hybrid micelle-based delivery systems, which also improved the survival rates of mice [74].

In addition to the aforementioned siRNA formulations, proteins and signaling pathway inhibitors have been applied in nanoparticle-based therapies for PADC. Francisco et al. co-loaded the Poly (ADP-ribose) polymerase1 (PARP-1) inhibitor olaparib (OLA) and ascorbic acid (AA) into calcium phosphate-based nanoparticles (NP-ACP-OLA-AA). The resulting formulation exhibited prolonged in vitro activity, heightened genotoxicity, and enhanced apoptosis-mediated cytotoxicity [75].

**Figure 3:**
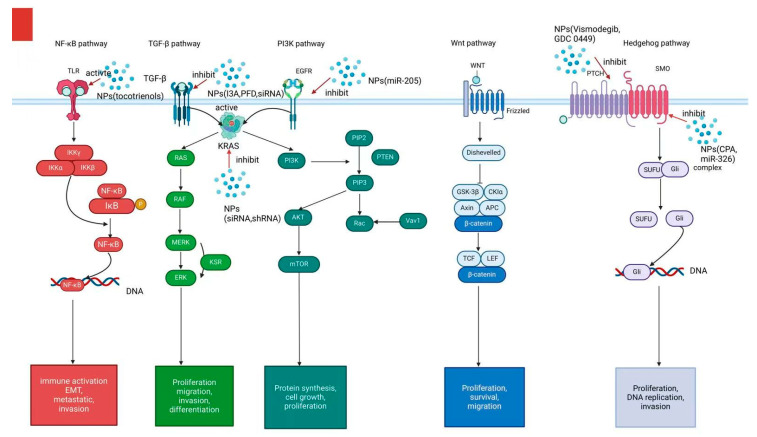
Several signaling pathways (e.g., NF-κB, TGF-β,PI3K, Wnt, and Hedgehog signaling) contribute to the proliferation, invasion, migration, and Epithelial-mesenchymal transition (EMT) of PDAC. Different NPs have been developed to target these signaling pathways and inhibit tumorprogression.

Cyclopamine (CPA), a naturally occurring steroidal alkaloid, inhibits the hedgehog (Hh) pathway by targeting smoothened (SMO) receptors (Figure 3), thereby enhancing tumor perfusion and facilitating the accumulation and distribution of nanomedicines [76]. Zhang et al. utilized PEG-poly (lactic acid) (PEG-PLA) as a delivery vehicle for CPA. Although no significant reduction in tumor size was observed compared to the control group, this treatment effectively inhibited the Hh pathway and reduced the potential risk of PDAC invasiveness and metastasis [50].

Erlotinib, an EGFR inhibitor, blocks cell cycle progression by inducing apoptosis through the upregulation of p27 and caspase activation, while inhibiting EGFR phosphorylation. This mechanism suppresses DNA synthesis, cell growth, and angiogenesis in PDAC cells [77,78,79]. Noorani et al. loaded erlotinib onto albumin nanoparticles (ANPs) and demonstrated significantly increased cytotoxicity after 72 h in vitro [52].

#### 3.1.2. Cytotoxic Chemotherapeutic Drugs

As a major component of the stroma of PDAC, CAFs represent a significant barrier to effective treatment of PDAC. To improve drug delivery through stromal depletion, a CAF-targeting nanopolymer, Cellax-docetaxel (DTX), was developed by conjugating DTX with acetylated carboxymethyl cellulose. Compared to the same dose of Nab-PTX, Cellax-DTX increased systemic drug exposure by 37 times and tumor uptake by 203-fold [80].

Chen et al. constructed an in vitro 3D PDAC model to evaluate the extracellular matrix (ECM) modulation capabilities of the GEM@nanogel (NGH) system. This system demonstrated robust ECM degradation, enhanced solid tumor penetration both in vitro and in vivo, and improved cytotoxicity by controlled GEM release and tumor cytoplasm disintegration [81]. The anticancer effects of doxorubicin (Dox) are often hindered by various drug resistance pathways, leading to poor treatment outcomes in PDAC. SiRNAs can reverse drug resistance by downregulating anti-apoptotic proteins when encapsulated into nanoparticles and co-delivered with siRNAs [82]. Thus, Hajar et al. used chitosan nanoparticles for dual delivery of insulin-like growth factor 1 receptor(IGFR1) siRNA and Dox, achieving favorable results in the A549 lung cancer cell line [83]. Given the high expression of IGFR1 in PDAC, we speculate that this therapeutic approach may also achieve favorable treatment outcomes in PDAC.

To addressing stromal barriers, a dual-drug delivery system combining the antifibrotic drug halofuginone (HF) and PTX was synthesized using methoxy polyethylene glycol-b-polycaprolactone. Pretreatment with HF nanoparticles restored stromal homeostasis by decreasing the major component of the ECM, substantially promoting PTX nanoparticle distribution, and penetrating cancer cells. This approach also improved cytotoxic T cell infiltration and resulted in significant tumor regression in two PDAC models [84].

Liu et al. explored the possibility of enhancing the chemotherapeutic efficacy of Mithramycin A (MIT) by encapsulating it into methoxy PEG-block-poly (d, l-lactic-co-glycolic acid) (mPEG-PLGA) NPs. By inhibiting the transcription factor Sp1, MITexhibited significantly better tumor suppression than free MIT (86% vs. 51%, *p* < 0.01) [51].

### 3.2. Nanoliposomal

Liposomes are double-layered lipid structures composed of phospholipids and cholesterol. Phospholipids form a bilayer structure that enhances the solubility and stability of anticancer drugs, facilitating effective drug encapsulation and delivery. Cholesterol, which is integrated within the bilayer, reduces the fluidity of the lipid membrane, thereby increasing the stability of nanoparticles in circulation. Additionally, it enhances the permeability of hydrophobic molecules across the plasma membrane, thereby improving drug transport and retention [68,85].

#### 3.2.1. Nanoliposome Irinotecan

Irinotecan exerts its anticancer effects by stabilizing the complex between topoisomerase I (TOP1) and DNA, leading to DNA strand breaks and inhibition of replication. To enhance therapeutic efficacy and reduce toxicity, Ko et al. formulated irinotecan into liposomal nanoparticles (nal-IRI), which prolonged the exposure time of the active metabolite SN-38 in tumor tissues while minimizing systemic toxicity. Clinical outcomes showed that patients receiving nal-IRI plus 5-FU/LV had significantly improved OS and PFS compared to those treated with 5-FU/LV alone (0.67 and 0.56, respectively) [86].

To further augment therapeutic efficacy, Wang et al. developed a co-loaded nanoparticle drug combined with irinotecan and the Hedgehog pathway inhibitor, GDC-0449. This combination not only suppressed the expression of tumor-associated markers, including collagen, α-smooth muscle actin (α-SMA), and GLI family zinc finger 1 (GLI-1), but also promoted apoptosis in tumor cells [53].

#### 3.2.2. Nab-PTX

Paclitaxel (PTX) enhances the efficacy of GEM by inhibiting tumor fibrosis and suppressing the expression of cytidine deaminase (CDA), an enzyme responsible for GEM inactivation [87]. However, systemic and repeated co-administration often leads to severe toxicity and limited therapeutic responses. Shabana et al. developed a thermosensitive, biodegradable hydrogel encapsulating PR_b-functionalized liposomes co-loaded with GEM and PTX for sustained local delivery to PDAC. The system exhibited prolonged drug release, enhanced cytotoxicity against PANC-1 cells, and greater tumor inhibition compared to non-targeted or free drug formulations [54].

Additionally, Yang et al. designed antibody fragment (AF)-conjugated liposomes encapsulating GEM and containing PTX (AF-GPL). Experimental results revealed an increased Bax/Bcl-2 ratio, demonstrating notable therapeutic effects in PDAC [55]. Similarly, Meng et al. used lipid-coated mesoporous silica nanoparticles (MSNP) for the co-delivery of PTX and GEM (LB-MSNP). Compared to free GEM, this system increased the active phosphorylated GEM metabolite by 13-fold, while reducing inactivated and deaminated metabolites by 4-fold, effectively inhibiting primary tumor growth and eliminating metastatic lesions [88].

In a clinical setting, Hingorani et al. conducted a randomized trial involving 279 patients with metastatic pancreatic cancer, evaluating polyethylene glycol-conjugated recombinant human hyaluronidase (PEGPH20) combined with nab-PTX/GEM. Combination therapy improved progression-free survival (PFS) with similar thromboembolic (TE) event rates compared to the control group [89]. Wei et al. designed a nanomedicine named TSL/HSA-PE, consisting of HSA complexes with PTX and ellagic acid (EA) co-encapsulated into thermosensitive liposomes (TSLs), which enhances drug retention and achieves better tumor accumulation and matrix penetration upon localized tumor heating [56].

#### 3.2.3. Targeted Therapy Drugs

Lipid-based nanomedicines can enhance the cellular uptake of drugs like RNA, improving targeting and transfection efficiency and promoting drug release after uptake by macrophages into tumor tissues to improve activity [86]. Rao et al. incorporated bifunctional short hairpin RNA (bi-shRNA) into nanoliposomes to target and silence *KRASG12mut*, inhibiting tumor growth with low toxicity and offering a novel PDAC treatment [90].

Nuclear factor kappa-B (*NF-κB*) plays an important role in PDAC growth and chemotherapy resistance. Tocotrienols may enhance GEM antitumor activity via *NF-κB* activation. Maniam et al. used smoke nanoparticles to encapsulate GEM and tocotrienol. This nanomliposamal formulation showed a 2.78-fold increase in the anti-proliferative effect of GEM when combined with tocotrienol [57].

Stimulator of Interferon Genes (STING) is an endoplasmic reticulum receptor that induces inflammation and activates antitumor activity [91]. Shaji et al. encapsulated the STING agonist 2′3′-cyclic guanosine monophosphate (2′3′-cGAMP) in nanoliposomes, achieving efficient drug delivery and tumor proliferation inhibition through STING signaling activation [38,58].

#### 3.2.4. Antifibrosis Drugs

Fibrotic stroma is a critical characteristic of the pancreatic tumor microenvironment. Therefore, Ji et al. synthesized a β-cyclodextrin (β-CD)-modified matrix metalloproteinase-2 (MMP-2) liposome, integrating antifibrosis agents and GEM for the treatment of PDAC. This approach demonstrated increased drug perfusion without severe adverse events [59].

### 3.3. Polymeric Micelles

Polymeric micelles are amphiphilic block copolymers with hydrophobic cores and hydrophilic shells for drug loading. These micelles have a high payload capacity, long circulation time, enhanced drug permeability, strong tumor penetration, and uniform distribution, making them a widely used nanocarrier system [92].

#### 3.3.1. Cytotoxic Chemotherapeutic Drugs

Cytotoxic chemotherapeutic drugs are crucial for PDAC treatment. Chen et al. designed a micelle with polyethylene glycol-polyarginine-polylysine to deliver mono-phosphorylated GEM and PTX, releasing PTX in the tumor’s acidic microenvironment to inhibit metastasis [93]. Additionally, Li et al. designed a fluorouridine (FUDR) micelle in combination with the tumor-penetrating peptide iRGD to further enhance anti-tumor effects [94].

#### 3.3.2. Targeted Therapy Drugs

Sonic hedgehog (SHH), the most studied member of the Hh signaling pathway, is commonly overexpressed in PDAC [95]. Kumar et al. designed polymer micelles to co-deliver miRNA targeting let-7b and vismodegib, a hedgehog pathway inhibitor. These micelles were demonstrated in serum for more than 24 h, with high uptake and low cytotoxicity, effectively inhibiting tumor growth [96]. Wang et al. used polymer micelles encapsulating irinotecan and vismodegib suppressing tumor growth and metastasis by inhibiting glioma-associated protein 1 and glucuronyl transferase expression [53]. Ray et al. created pH-responsive micelles with GEM and the Hh inhibitor (GDC-0449), selectively releasing contents in the acidic PDAC microenvironment and inhibiting tumor proliferation [62]. Daman et al. loaded salinomycin (SAL) into PEG-PLA micelles, inhibited invasion, and harnessed EMT in cancer stem cells (CSCs), achieving therapeutic efficacy in Balb/c AsPC-1 xenograft mice [97].

Additionally, nanomicells targeting various molecular targets in tumors have been developed. Pittella et al. constructed a nano-micelle (PEG-CCP/CaP) composed of polyethylene glycol and calcium phosphate, designed to deliver siRNA targeting VEGF. After intravenous administration, this drug demonstrated a 70% gene silencing efficiency in the BxPC3 pancreatic tumor model, significantly inhibiting tumor growth [98]. In PDAC, the oncogene microRNA-34a (*miR-34a*) is notably downregulated and targets various oncogenes involved in tumor proliferation, apoptosis, and invasiveness. High expression levels of PLK1 are strongly correlated with poor short-term survival in patients with pancreatic cancer. In response, Xin et al. engineered a reactive oxygen species (ROS)-responsive polymer micelle to co-deliver the PLK1 inhibitor volasertib and miR-34a. This drug delivery system significantly inhibited tumor growth while minimizing systemic toxicity [64].

#### 3.3.3. Immunomodulators

Ingenol-3-mebutate (I3A) acts as a novel immunomodulator by upregulating CD80 and CD86 expression on dendritic cells (DCs), which in turn activates CD8+ T cells and induces mitochondrial swelling, leading to tumor cell death. Yu et al. developed an I3A-loaded polymer micelle (I3A-PM) that promotes Th1 polarization. By upregulating Th1 cytokines (IL-12, IL-2, IFN-γ, and TNF-α), I3A-PM accelerates the expansion of CD4+ and CD8+ T cells and inhibits TGF-β signaling which leads to regulatory T cell and Th2 cytokine IL-6 depletion, ultimately suppressing tumor proliferation and metastasis [99]. Furthermore, Shen et al. created a nano-micelle system containing I3A modified with 2-3-((S)-5-amino-1-carboxy-pentyl)-ureido)-pentanedioate (ACUPA-) and triphenylphosphine (TPP+). This formulation significantly increased the infiltration of T lymphocytes into the tumor, activated adaptive immunity, and induced immunogenic cell death, achieving promising therapeutic effects [63].

#### 3.3.4. Combination Drugs

Zhao et al. loaded CPA and PTX onto micelles to regulate CAFs in PDAC stroma. This nanomedicine was found to disrupt the communication between tumor cells and CAFs, breaking the cycle of tumor cell proliferation, stromal support, and metastasis [100]. Li et al. developed a nano-polymer micelle (M-CPA/PTX) for co-deliver CPA and PTX, which enhanced the intratumoral vasculature density, promoted CD8+ T cell infiltration, without depletion of tumor-restraining stroma [60]. GEM, a first-line PDAC treatment, was developed by Mondal et al. using a cetuximab (C225)-decorated micelle targeting EGFR, effectively improving drug accumulation in pancreatic tumor-bearing mice and suppressing tumor growth [61].

Resistance to GEM in PDAC is often associated with the activation of the STING signaling pathway, which induces chemokines like CCL2 and CCL7 [101]. This process leads to immune resistance by recruiting tumor-associated macrophages (TAM) and myeloid-derived suppressor cells (MDSC). To address this, Wan et al. incorporated the CCR2 antagonist PF-6309 (PF), which shares receptors with CCL2 and CCL7, into a GEM-conjugated polymer (PGEM) micelle system. This approach not only reduces the pancreatic tumor burden but also induces effective anti-tumor immunity [91].

### 3.4. Inorganic Nanoparticles

Inorganic nanoparticles have emerged as promising drug carriers, offering improved therapeutic efficacy and reduced side effects. Their advantages include simplified modification of target molecules, control over the drug release rate through different stimuli, and efficient delivery of targeted drugs. Common materials used in inorganic nanoparticles include gold, silica, and iron oxide [102].

#### 3.4.1. AuNPs

Han et al. designed AuNPs coated with PEI and PEG to co-deliver all-trans retinoic acid (ATRA, which promotes PSC quiescence) and siRNA targeting heat shock protein 47 (HSP47, a collagen-specific chaperone). This nanoparticle system promotes PSC quiescence and suppresses ECM hyperplasia, thereby facilitating drug delivery and accumulation in pancreatic tumors and significantly boosting the effectiveness of chemotherapy agents [65].

The expression of SMO in the Hh pathway is regulated by various microRNAs, including miR-193b, miR-326, and miR-338-3p. Based on this, Mo et al. designed gold nanoparticles to carry miR-326. Although the study was conducted in hepatocellular carcinoma, the Hh pathway is also expressed in PDAC, suggesting the potential therapeutic relevance of this approach in PDAC treatment [66].

#### 3.4.2. Iron Oxide NPs

Deby et al. conjugated relaxin 2 (RLX) with superparamagnetic iron oxide nanoparticles to target CAF precursors. This strategy not only delayed tumor growth but also enhanced anti-tumor effects when combined with GEM [103].

IGFR1 is highly expressed in 40–90% of PDAC tissues, as well as in both tumor and stromal cells, making it a potential biomarker for targeting tumor tissues. Zhou et al. developed magnetic iron oxide nanoparticles conjugated with recombinant human IGF1 and Dox. This nanosystem inhibited the proliferation of tumor tissues and enabled treatment response monitoring using MRI [104].

#### 3.4.3. Tungsten Oxide NPs

Activation of the PI3K pathway can promote tumor invasion, migration, EMT, and drug resistance [105]. Huo et al. designed a nanoaggregated system using tungsten oxide nanoparticles modified with the hypoxia-directed chemokine CCL-28 ligand and MMP-2 cleavable peptides. This approach enables drug penetration into the deeper regions of tumors by targeting the hypoxic tumor microenvironment and alleviating hypoxic-induced resistance in a PI3K-dependent manner [106].

## 4. Summary and Future Directions

PDAC remains one of the most lethal cancers due to its late diagnosis, aggressive tumor biology, and profound resistance to conventional therapies. Nanomedicines have played a revolutionary role in addressing these challenges by enhancing diagnostic sensitivity, improving drug delivery efficiency, and overcoming TME-mediated therapeutic resistance.

From a diagnostic perspective, various nanoprobes—such as AuNPs, SPIONs, and BNTs—have been developed to target specific biomarkers, including CA19-9, claudin-4, and SMO, thereby improving early detection through enhanced imaging modalities like MRI, PET/CT, and NIR fluorescence imaging. These nanoparticles not only increase imaging sensitivity but also allow real-time monitoring of treatment responses. However, most diagnostic applications remain in the preclinical stages, with limited translation to clinical practice due to concerns about long-term toxicity, biodistribution, and regulatory approval processes.

The primary challenge in treating PDAC lies in its dense fibrotic stroma, which hinders perfusion and limits the penetration of chemotherapeutic agents. To overcome this obstacle, nanomedicine-based technologies have been developed to improve drug delivery and accumulation within tumor tissues via the EPR effect, while enabling controlled drug release through stimuli-responsive systems. Co-delivery platforms, such as micelles co-loaded with GEM and PTX or polymeric nanoparticles encapsulating both siRNA and chemotherapeutic agents, have demonstrated the ability to overcome drug resistance, remodel the fibrotic stroma, and enhance immune cell infiltration into the tumor microenvironment. Additionally, strategies targeting the TME plays an important role in chemotherapy resistance. ECMs driven by CAFs and aberrant oncogenic signaling pathways, such as Hh, enhances drug penetration and reduces stromal barriers. Approaches such as using relaxin-loaded nanoparticles to modulate CAFs or dual-drug systems to inhibit Hh signaling have shown promise in disrupting the ECM and enhancing drug accumulation within tumors. Research shows that inhibiting the Hh signaling pathway can reduce stromal barriers and improve perfusion, ultimately facilitating more effective drug delivery. Despite these advances, the heterogeneity of the TME, variability in the EPR effect among patients, and potential immunogenicity of certain nanocarriers remain hurdles.

One critical limitation of the clinical translation of nanomedicines is their complex manufacturing processes, scalability, and reproducibility. Furthermore, off-target accumulation and potential toxicity, especially for inorganic nanoparticles such as Au NPs and tungsten oxide nanoparticles, raise safety concerns that require thorough investigation. Moreover, single nanomedicine treatments are not effective in clinical settings and are typically used in combination with other chemotherapy drugs, which inevitably leads to further toxic adverse effects. The pharmacokinetics and long-term fates of these nanomaterials require comprehensive evaluations to ensure patient safety. Other technologies like interventional techniques, together with NPs, have been successfully applied clinically for the effective treatment of locally advanced PDAC, but their role in the metastasis stage is limited. Future research should focus on developing personalized nanomedicine approaches that account for interpatient variability in TME characteristics and nanoparticle uptake. The combination of nanomedicine with immunotherapy, targeted therapy, and radiotherapy could further potentiate therapeutic efficacy. In addition, the design of multi-functional nanoplatforms capable of simultaneous diagnosis, drug delivery, and real-time monitoring holds great promise. In recent years, TME-responsive nanoplatforms have demonstrated promising results in multimodal imaging-guided therapies, including photodynamic, photothermal, and chemodynamic treatments for various tumors. These nanoplatforms selectively release drugs or activate therapeutic functions in response to TME characteristics, resulting in enhanced selectivity, minimal side effects, and strong control. Furthermore, magnetic micro-robots that target folate receptors offer an innovative therapeutic approach that harness the properties of magnetic fields combined with folate receptor specificity. When integrated with imaging modalities, these microrobots enable real-time monitoring of drug delivery and treatment progression, significantly improving the precision of drug administration. To facilitate clinical translation, collaborative efforts between researchers, clinicians, and regulatory agencies are essential to establish standardized protocols for safety assessment and efficacy evaluation.

## 5. Conclusions

In conclusion, while nanotherapy has shown remarkable potential for improving the diagnosis and treatment of PDAC, addressing the current limitations and ensuring safe, effective, and patient-tailored solutions are critical for its successful clinical application.

## Figures and Tables

**Table 1:** Nanomaterials used for laboratory tests.

Biomarker	Nanomaterials	Sample	Sources	Outcomes	Reference
CA19-9	polythionine-Au composites	Serum	Human	a detection limit of 0.26 U/mL at a signal-to-noise ratio of 3	[13]
CEA	a novel enzyme-labeled gold nanoparticle probe	Serum	Human	a detection limit of 12 ng/L	[14]
MUC4	Au NP	Serum	Human	a high sensitive detection of a single AuNP	[15]
miRs	AuNP with a localized surface plasmon resonance sensor	Plasma	Human	allowed for the simultaneous measurement of multiple miRs	[16]
miRs	α-hemolysin (αHL) single nanopore	miRNA reagents	_	distinguished complex molecular signals of miR-21, miR-155, and miR-196a	[17]
Evs	semiconductor nanomaterial-based rapid fluorescence immunoassay	Serum	Human	improved the ability of serum levels of EV EpCAM and EV EphA2	[18]
Evs	lternating current (AC) electrokinetic microarray chip	Whole blood, serum, or plasma	Human	a detection of glypican-1 and CD 63 with 99%sensitivity and 82%specificity	[19]
Exosomes	polydopamine (PDA)-modified slide	Serum	Human	a detection limit of 9 × 10^−19^ mol/L, differentiated stage I-II from stage III tumors with a sensitivity of 95.7%	[20]
Regenerating Family Member 1 Alpha	nanobiotinylated liposome based on isothermal amplification	Urine	Human	a detection limit of 1 fg/mL	[21]
CA19-9	screen-printed interdigitated electrodes immobilized with anti-CA19-9 antibodies	Whole cell lysates of colorectal adenocarcinoma	HT-29 cell lines, SW-620 cell lines	a detection limit of 0.12 U/mL	[22]
claudin-4	nanoprobes based on modified apoferritin (mAFTN), functionalizing CdSe (ZnS), Quantum dots(QDs)	Cells	Capan-1 cells	a detection sensitivity 27 times higher than conventional organic fluorophores and 6 times higher than individual QDs	[20]
FAP	AuNPs with heterobifunctional polyethylene glycol (PEG)	Tissues	Human	labeled tumor stroma in approximately 5 µm thick	[23]

**Table 2:** Nanomaterials for imaging examinations.

Imaging	Nanosystems	Targeting Molecular	Cell Lines	In Vivo Models	Outcomes	Reference
NIR imaging	bMSN@Cy7.5-FA NPs	Folate receptors	A549, HEPG2 and BxPC-3 cell lines	BxPC-3 orthotopic mouse model	reached a maximum at a post injection time-point of 12 h	[28]
NIR imaging	CR@E8-EVs	CDH17	MKN45 and 4T1 cells	MKN45 orthotopic mouse model	exhibited strong NIR absorption and remarkable active tumor targeting capability	[29]
NIR imaging	AP1153-ICG-NJs	CCK-BR	Panc-1 cells	Panc-1 orthotopic mouse model	facilitated delivery of NPs tumors in vivo	[30]
CT imaging	AuNPs	_	PK-1, PK-45, and Panc-1 cells	_	showed preferential uptake by PDAC cells at an optimal diameter of 20 nm	[31]
MR imaging	Lip-Gd@AuNPs	_	_	C57 black mice model	identificated the pancreas with contrast-to-noise ratios exceeding 35:1	[32]
MR imaging	CXCR4-USPIO	CXCR4	AsPC-1, BxPC-3, CFPAC-1, and Panc-1	_	semi-quantitatively assessed the cellular CXCR4 expression levels	[33]
MR imaging	Dex-g-PCL/SPIO nanoparticles	enolase 1	CFPAC-1 cell lines, MiaPaCa-2 cell lines	CFPAC-1 xenograft mouse model	enhance the detection of PDAC by in vivo and in vitro MRI	[34]

**Table 3:** Nanomedicines for the treatment of PDAC.

Naoncarriers	Polymers	Targeting Molecule	Drugs	Cell Lines	In Vivo Models	Outcomes	Reference
Polymeric NPs	poly(dimethylaminoethyl methacrylate)	SLC7A11	SLC7A11 siRNA	mouse KPC PDAC cells, human CAFs	KC (Kras-mutated) and KPC (Kras- and p53-mutated) mouse models	reduced CAF activation, inhibited tumor growth and metastases	[47]
Polymeric NPs	iRGD TPNs with polyethylene glycol (PEG)-peptide	KRAS	KRAS siRNA	KPC-derived cell lines, MIA PaCa-2 cell lines	MIA PaCa-2 transgenic pancreatic cancer mouse models	enhanced tumor-penetrating ability, inhibit tumor growth	[48]
Polymeric NPs	polyethylenimine	TGF-β	TGF-β1 siRNA	PaCa-2 cell lines	PaCa-2 orthotopic pancreatic cancer transplant model	prolong survival, break tumor-induced CD8(+) T cell suppression	[49]
Polymeric NPs	PEG-poly (lactic acid)	SMO	CPA	Capan-2 cell lines	Capan-2 xenograft mouse models	improved tumor perfusion, inhibited tumor growth	[50]
Polymeric NPs	mPEG-PLGA	transcription factor Sp1	MIT	BxPC-3 cells, MIA Paca-2 cells	BxPC-3 xenograft mouse models	enhanced tumor-penetrating ability, inhibit tumor growth	[51]
Polymeric NPs	albumin nanoparticles	EGFR	erlotinib	ASPC-1 cell lines, PNAC-1 cell lines	_	reduced durgs dose for killing cells	[52]
Nanoliposomal	PEG	TOP1	smart SN38 and GDC-0449	PSCs, BxPC-3 cells and MIA PaCa-2 cells	_	reduced tumor stroma, inhibited tumor growth	[53]
Nanoliposomal	poly(δ-valerolactone-co-D,L-lactide)-b-poly(ethylene glycol)-b-poly(δ-valerolactone-co-D,L-lactide)	_	PTX and GEM	PANC-1 cell lines	_	reduced toxic side effects, inhibited tumor gorwth, prolonged drug release	[54]
Nanoliposomal	1,2- distearoyl-sn-glycero-3-phosphoethanolamine-N-[methoxy(polye thylene-glycol)-2000] and maleimide	_	PTX and GEM	BxPC3 cells	_	increased cytotoxic effect in pancreatic cancer cell, inhibited tumor growth	[55]
Nanoliposomal	HAS	_	PTX and EA	HPaSteC cell lines, BxPC-3 cell lines	BxPC- and HPaSteC-bearing nude mouse model	enhanced tumor accumulation and matrix penetration	[56]
Nanoliposomal	smoke nanoparticles	NF-κB	GEM and tocotrienols	Panc 10.05, SW 1990, AsPC-1 and BxPC-3 cells	_	increased the anti-proliferative effect of GEM	[57]
Nanoliposomal	cGAMP LNPs	an endoplasmic reticulum receptor STING	2′3′-cGAMP, a high-affinity endogenous ligand of STING	Panc02 cells, NIH-3T3 cells, Human Pancreatic Duct Epithelial Cells, DC2.4 cells	Panc-02 and NIH-3T3 xenograft mouse models	increased the cellular uptake of 2′3′-cGAMP	[58]
Nanoliposomal	N-hydroxysuccinimide (NHS)/1-ethyl-3-[3-(dimethylamino)propyl] carbodiimide hydrochloride	PSCs	β-CD and MMP-2	PSCs, Panc-1 cells	pancreatic coimplanted tumor model	increased drug perfusion, decreased the stromal barrier	[59]
Micelles	poly(ethylene glycol) with poly(ε-caprolactone) and pendent quaternary ammonium cations	SHH	CPA and PTX	Kras * cells	Kras * orthotopic murine models	enhanced tumor infiltration of CD8+ T cells, prolong the survival time	[60]
Micelles	malemido-poly(ethylene glycol)-block-poly(2-methyl-2-carboxyl-propylene carbonate-graft-dodecanol	C225	GEM and miR205	MIA PaCa-2R cells	Miapaca-2 orthotopic mouse models	enhanced tumor accumulation of C225-micelles, increased apoptosis and reduced EMT	[61]
Micelles	PEG-b-poly (carbonate)	Hh pathway	GEM and GDC-0449	BxPC-3 cell lines, Mia PaCa-2 cell lines	BxPC-3 xenografts mouse models	suppressed tumor proliferation	[62]
Micelles	2-3-((S)-5-amino-1-carboxy-pentyl)-ureido)-pentanedioate (ACUPA-) and tri-phenylphosphine (TPP+)	_	I3A	MIAPaCa-2 cells, Panc02 cells	Panc02 PC orthopic mouse models	activated adaptive immunity, prolonged the survival time	[63]
Micelles	poly(ethylene glycol)-poly[aspartamidoethyl(p-boronobenzyl)diethylammonium bromide] (PEG-B-PAEBEA)	Bcl-2	miR-34a mimic, PLK1 inhibitor volasertib (BI6727)	MIA PaCa-2R cell line	NSG mice harboring orthotopic pancreatic tumors model	inhibited tumor growth	[64]
Au NPs	PEGylated polyethylenimine-coated gold nanoparticles	PSCs	all-trans retinoic acid and siRNA targeting heat shock protein 47	Panc-1 cell lines, Mia-PaCa-2 cell line	Panc-1 and PSCs xenograft tumour model	induced PSC quiescence and inhibited ECM hyperplasia	[65]
Iron oxide NPs	superparamagnetic iron oxide nanoparticle	CAFs	human relaxin-2	Primary human pancreatic stellate cells	Panc-1 and hPSCs orthotopic tumor model	inhibited tumor growth	[66]

* Kras cells exhibit a doxycycline-driven mutation of Kras^G12D^ oncogene.

## Data Availability

Not applicable.

## Abbreviations

The following abbreviations are used in this manuscript:

PDAC	Pancreatic ductal adenocarcinoma
NPs	nanomedicines
EPR	Enhanced permeability and retention
GEM	gemcitabine
5-FU	5-fluorouracil
LV	leucovorin
CA19-9	carbohydrate antigen 19-9
CT	computed tomography
MRI	magnetic resonance imaging
PET	positron emission tomography
US	Ultrasound
AuNPs	gold nanoparticles
CEA	carcinoembryonic antigen
ssDNA	single-stranded DNA
HRP	Horseradish peroxidase
SERS	Surface-enhanced Raman scattering
miRs	microRNAs
LSPR	localized surface plasmon resonance
αHL	α-hemolysin
EV	Extracellular vesicle
QD	Quantum dots
EphA2	Erythropoietin-producing hepatocellular A 2
EpCAM	Epithelial cell adhesion molecule
AC	Alternating current
MIF	macrophage migration inhibitory factor
GPC1	Glypican1
EGFR	Epidermal growth factor receptor
LAMP	liposome based on isothermal amplification
REG1A	Regenerating Family Member 1 Alpha
PCR	Polymerase chain reaction
SPIDE	screen-printed interdigitated electrode
CNO	carbon nano-onions
GO	graphene oxide
mAFTN	modified apoferritin
ZnS	CdSe
Ni-NTA	nickel-nitrilotriacetic acid
PEG	polyethylene glycol
FAP	fibroblast activation protein
NIR	near-infrared
PAI	photoacoustic imaging
FA	folic acid
CR	croconaine
CDH17	Cadherin 17
HSA	human serum albumin
CCK-B	cholecystokinin B
ICG	indocyanine green
CPSNP	calcium phosphate silica nanoparticles
Bi	bismuth
Pt	platinum
BNTs	bismuth subcarbonate nanotubes
SPIONs	superparamagnetic iron oxide nanoparticles
NSF	nephrogenic systemic fibrosis
CXCR4	Chemokine receptor 4
ENO1	enolase 1
^18^F	18F-fluorodeoxyglucose
KRAS	Kirsten rats arcomaviral oncogene homolog
IGFR1	insulin-like growth factor 1 receptor
TGF-β	transforming growth factor beta
OS	overall survival
CAFs	cancer-associated fibroblasts
PSCs	pancreatic stellate cells
TAMs	regulatory T cells, tumor-associated macrophages
TME	tumor microenvironment
EMT	Epithelial-mesenchymal transition
siRNA	small interfering RNA
iRGD	internalizing RGD peptide
PDCD4	Programmed cell death protein 4
PTEN	Phosphatase and tensin homolog deleted on chromosome ten
TPM1	Tropomyosin 1
PEI	polyethylenimine
Nab-PTX	Nab-paclitaxel
Hh	hedgehog
CPA	Cyclopamine
SMO	smoothened
DTX	docetaxel
NGH	nanogel
Dox	doxorubicin
ANPs	albumin nanoparticles
HF	halofuginone
OLA	olaparib
AA	ascorbic acid
nal-IRI	nanoparticles
α-SMA	α-smooth muscle actin
PTX	Paclitaxel
CDA	cytidine deaminase
AF	antibody fragment
PFS	progression-free survival
TE	thromboembolic
EA	ellagic acid
TSLs	thermosensitive liposomes
bi-shRNA	bifunctional short hairpin RNA
NF-κB	Nuclear factor kappa-B
MIT	Mithramycin
STING	Stimulator of Interferon Genes
β-CD	β-cyclodextrin
MMP-2	matrix metalloproteinase-2
MDSC	myeloid-derived suppressor cells
FUDR	fluorouridine
SHH	Sonic hedgehog
SAL	salinomycin
CSCs	cancer stem cells
I3A	Ingenol-3-mebutate
DC	dendritic cell
TPP+	triphenylphosphine
ROS	reactive oxygen species
ATRA	all-trans retinoic acid
RLX	relaxin 2
